# NK Cells Equipped With a Chimeric Antigen Receptor That Overcomes Inhibition by HLA Class I for Adoptive Transfer of CAR-NK Cells

**DOI:** 10.3389/fimmu.2022.840844

**Published:** 2022-05-02

**Authors:** Xiaoxuan Zhuang, Eric O. Long

**Affiliations:** Laboratory of Immunogenetics, National Institute of Allergy and Infectious Diseases (NIAID), National Institutes of Health (NIH), Rockville, MD, United States

**Keywords:** natural killer cell, chimeric antigen receptor, immunotherapy, inhibitory receptor, tumor cell

## Abstract

Dominant inhibitory receptors for HLA class I (HLA-I) endow NK cells with high intrinsic responsiveness, a process termed licensing or education, but hinder their ability to kill HLA-I^+^ tumor cells. Cancer immunotherapy with adoptive transfer of NK cells must overcome inhibitory signals by such receptors to promote elimination of HLA-I^+^ tumor cells. As proof of concept, we show here that a chimeric antigen receptor (CAR) can be engineered to overcome inhibition by receptors for HLA-I and to promote lysis of HLA-I^+^ tumor cells by CAR-NK cells. The design of this NK-tailored CAR (NK-CAR) relied on the potent NK cell activation induced by the synergistic combination of NK receptors CD28H (CD28 homolog, *TMIGD2*) and 2B4 (CD244, *SLAMF4*). An NK-CAR consisting of the single-chain fragment variable (scFv) of a CD19 antibody, the CD28H transmembrane domain, and the fusion of CD28H, 2B4, and TCRζ signaling domains was compared to a third-generation T-cell CAR with a CD28-41BB-TCRζ signaling domain. The NK-CAR delivered stronger activation signals to NK cells and induced more robust tumor cell lysis. Furthermore, such CAR-NK cells could overcome inhibition by HLA-E or HLA-C expressed on tumor cells. Therefore, engineering of CAR-NK cells that could override inhibition by HLA-I in patients undergoing cancer immunotherapy is feasible. This approach offers an attractive alternative to more complex strategies, such as genetic editing of inhibitory receptors in CAR-NK cells or treatment of patients with a combination of CAR-NK cells and checkpoint blockade with antibodies to inhibitory receptors. A significant benefit of inhibition-resistant NK-CARs is that NK cell inhibition would be overcome only during contact with targeted tumor cells and that HLA-I on healthy cells would continue to maintain NK cell responsiveness through licensing.

## Introduction

Immunotherapy with CAR-T cells has been very successful for clinical treatment of hematologic cancers (1). However, adverse side-effects associated with infusion of CAR-T cells, such as severe cytokine-release-syndrome (CRS) and neurotoxicity, could be life-threatening and need to be carefully managed (2). Natural killer cells might be preferable for adoptive cell therapy because of their safer cytokine profile and low graft-versus-host-disease (GVHD) activity in hematopoietic stem cells transplantation (3, 4). To reduce the cost of CAR-T cell therapy and to simplify the process of manufacturing CAR-T cells, “off-the-shelf” CAR-T strategies have been proposed (1). T cell receptors (TCRs) on allogenic CAR-T cells that derived from healthy donors can be silenced to minimize GVHD and to produce universal cryopreserved CAR-T products, which can be used when needed (2). Notably, unlike T cells, “off-the-shelf” CAR-NK strategies can be developed without the need to genetically silence a receptor (5, 6). Safety and persistence of donor-derived NK cells have been demonstrated in clinical hematopoietic stem cells transplantation (7). Moreover, NK germline-encoded receptors for tumor ligands can contribute additional beneficial effects.

Activation of primary NK cells requires synergistic combination of signals from activation receptors, the ligands of which are usually upregulated on transformed or infected cell (8, 9). However, activation signals alone may not be sufficient for an NK cell response, as NK cell activation is controlled by inhibitory receptors for HLA-I, including receptor NKG2A-CD94 for the non-classical HLA-I molecule HLA-E and the inhibitory killer-cell Ig-like receptors (KIR) for classical HLA-I molecules, specifically, KIR2DL1 for HLA-C group 2 (C2) allotypes, KIR2DL2/3 for HLA-C group 1 (C1) allotypes, and KIR3DL1 for certain HLA-B and HLA-A allotypes (10). Therefore, adoptive cell therapy using NK cells must take into account inhibitory signals of NK receptors for HLA-I molecules on tumor cells, which have to be overridden in order to achieve optimal NK-cell activation. This could be achieved by checkpoint blockade of NKG2A (11). Alternatively, silencing of NKG2A or KIRs in CAR-NK cells has been proposed (12). Here we show that an NK-tailored CAR (NK-CAR), combining the signaling domains of CD28H, 2B4, and TCRζ, provided strong activation signals to synergistic NK cells. Compared to third-generation T-cell CARs (T-CAR, CD28-41BB-TCRζ), which have been used in NK cells, our NK-CAR (CD28H-2B4-TCRζ) induced a more robust anti-tumor cytotoxic activity in NK cells and was more potent in overcoming inhibition. Our results support the design of tailored NK-CARs as a superior approach to the use of T-CARs in order to induce inhibition-resistant NK-cell activation. Such an approach has the potential to simplify CAR-NK therapy, as it would eliminate the need to silence inhibitory receptors or the use of checkpoint blockade.

## Materials and Methods

### Cells

Human NK cells were isolated from peripheral blood of healthy donors by negative selection using the EasySep™ NK cell isolation kit (Stemcell Technologies). Freshly isolated, unstimulated (referred to here as resting) NK cells were resuspended in Iscove’s modified Dulbecco’s medium (IMDM; Gibco) supplemented with 10% human serum (Valley Biomedical) and used within 4 days. The cell line NKL was obtained from M.J. Robertson (Indiana University Cancer Research Institute, Indianapolis, IN) and cultured in IMDM with 10% heat-inactivated fetal calf serum (FCS, Gibco). P815 cells (ATCC), 721.221 cells (referred to here as 221 cells), and HLA-E or HLA-C (Cw15, group C2) transfected 221 cells were cultured in RPMI-1640 (Gibco) supplemented with 10% heat-inactivated FCS.

### Plasmids and Lentivirus Production

CAR constructs were either synthesized as gBlocks (IDT) or amplified by PCR from existing templates, and cloned into the EcoRI and NotI restriction sites of the pCDH-EF1-T2A-Puro vector (System Biosciences). All plasmid constructions were performed using the In-Fusion HD cloning kit (Clontech) and verified by DNA sequencing. For production of lentivirus, low-passage Lenti-X 293T cells (Clontech) were transfected with DNA using the PEI Max 40K (polyethyleneimine) technique, as described (8). Supernatant from Lenti-X 293T cell cultures was collected 2 days after transfection and passed through a 0.45 μM filter. Culture media were used for transduction either directly or concentrated using PEG*-it* (System Biosciences).

### Cytotoxicity Assay

Calcein-AM release assays were used to determine lysis of target cells by NK cells (13). Briefly, target cells at a final concentration of 10^6^/ml in complete media were incubated with 15μM calcein-AM (Invitrogen) at 37°C for 30 min. After 2 washes with complete media, labeled target cells were added to 96-well-plate at 10^4^/well. Effector NK cells were added at different effector to target (E:T) ratios and incubated for 4 hours. Target cell lysis was determined by the fluorescence of calcein-AM released in the supernatant and measured with a plate reader (Enspire, Perkin Elmer, MA and SpectraMax plus, Molecular Devices, CA).

### Stimulation of NK Cells With Beads

Cells were stimulated with antibody-coated goat-anti-mouse Dynabeads (Invitrogen) at the ratio of 4 beads per cell. Antibodies used for bead coating include Myc-tag antibody (9B11, mouse IgG2a, Cell Signaling), NKG2A antibody (Z199, mouse IgG2b, Beckman Coulter), control mouse IgG2b (MOPC-141, Sigma), and control mouse IgG2a (UPC-10, Sigma). 10^7^ beads were coated for 30 min at 37°C with 2 μg total IgG at different combinations: 1 μg mIgG2a + 1 μg mIgG2b, 1 μg mIgG2a + 1 μg anti-NKG2A, 1 μg anti-Myc-tag + 1 μg mIgG2b, or 1 μg anti-Myc-tag + 1 μg anti-NKG2A. Beads were washed with hank’s-balanced-salt-solution (HBSS, Corning) in 1% FCS to remove excessive antibodies and resuspended in the same buffer. Pre-chilled beads and cells were mixed on ice and incubated in a 37°C water bath for 10 min. Cells were washed and lysed before analyzing by western blot using phospho-Erk1/2 (Cell Signaling #9101) and phospho-PLCγ1 (Cell Signaling #2821) antibodies.

### Data Sharing Statement

All supporting data are available on request.

## Results

**Figure 1:**
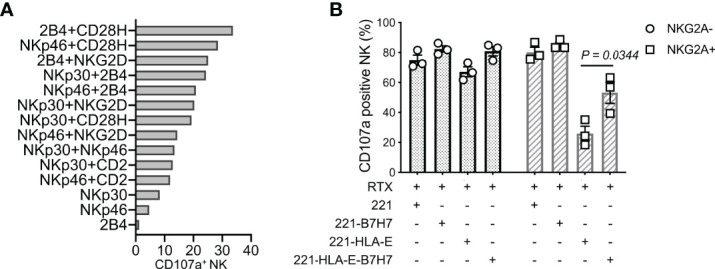
NK cell degranulation triggered by the synergy of receptors CD28H and 2B4. **(A)** Degranulation of resting human NK cells induced by crosslinking combinations of NK-cell activating receptors. Crosslinking was performed in a redirected cytotoxicity assay using P815 cells in the presence of antibodies for the indicated activating receptors (n=2). **(B)** Degranulation of resting NK cells in the presence of rituximab (RTX) and untransfected 221 cells or transfected 221 cells that express B7H7, HLA-E, or both.

Synergistic combinations of NK activation receptors were tested in a redirected cytotoxicity assay with mouse CD32**^+^** P815 cells and antibodies to 6 activation receptors. The pairing of CD28H and 2B4 induced a particularly robust NK-cell activation and degranulation (**Figure 1A**). To test how the natural ligand of CD28H, B7H7 (HHLA2), contributes to NK cell activation in the context of inhibition by HLA class I, HLA-I-negative 221 cells were transfected with HLA-E and B7H7. Although 221 cells express several ligands of NK cell activation receptors, including CD48 for 2B4 and CD58 for CD2, 221 cells do not elicit a strong response of primary resting NK cells. As B lymphoblast cells, such as 221, express CD20, the CD20 mAb rituximab was used to stimulate NK cells through CD16 and obtain a stronger response. Despite the engagement of multiple NK receptors, including CD16, 2B4, and CD2, NKG2A+ NK cells were inhibited by HLA-E expressed on 221 cells, while NKG2A-negative cells in the same NK population were not (**Figure 1B**). Remarkably, B7H7 expression on 221-HLA-E cells was sufficient to restore a significant NK cell response, as measured by degranulation. The result indicated that CD28H can further enhance NK cell activation and promote resistance to inhibition while receptors 2B4 and CD16 are both engaged.

**Figure 2:**
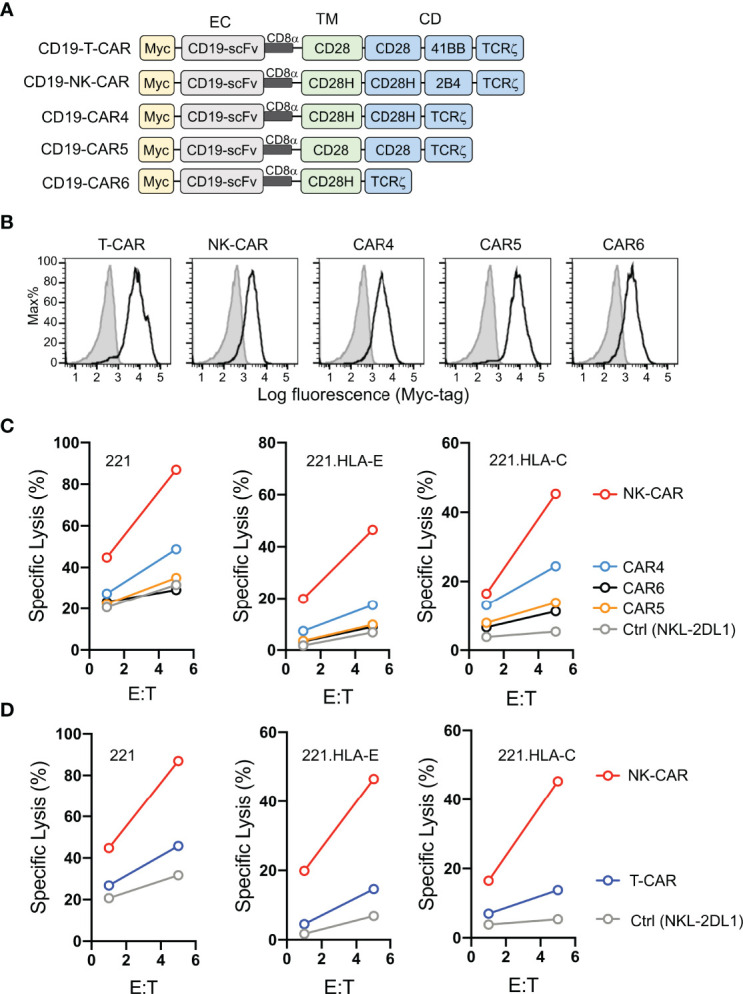
Lysis of CD19^+^ HLA-I^+^ tumor cells by NK cells that express CD19.CARs. **(A)** Schematic diagram of CAR constructs using anti-CD19 scFv with a N-terminal myc-tag as extracellular domain and the indicated combinations of signaling domains. **(B)** Histograms showing expression of CAR constructs in NKL.2DL1 cells. **(C)** Killing assays comparing lysis of target cells by the indicated CAR-expressing NKL.2DL1 cells. The HLA-A, HLA-B and HLA-C negative lymphoblastoid cell line 221, or 221 cells transfected with HLA-E or HLA-Cw15 were used as target cells (n=3). **(D)** Comparing target-cell lysis by NKL.2DL1 cells expressing NK-CAR or T-CAR (n=3). Same target cells were used as in **(C)**.

Having shown that fusion of full-length CD28H with the TCRζ cytoplasmic domain results in potent NK cell activation (8), and that CD28H and 2B4 form a strong synergistic pair of activation receptors (**Figure 1A**), we reasoned that inclusion of the signaling domain of 2B4 in the chimeric CD28H-TCRζ cytoplasmic domain would induce even greater NK cell activation, which might surmount strong inhibitory signals. We tested it in the context of a CAR using the CD19 scFv with a Myc-tag as extracellular domain for targeting CD19^+^ cancer cells. Several combinations of transmembrane and signaling domains were tested and compared to a third generation T-cell CAR consisting of the transmembrane and cytoplasmic domain of CD28 fused to the 4-1BB (CD137, *TNFRSF9*) and TCRζ cytoplasmic domains (**Figure 2A**). A T-CAR with a CD28-TCRζ cytoplasmic domain was also constructed. A CD19 scFv CAR version of our CD28H-TCRζ chimera was made and compared with a version having only the TCRζ cytoplasmic domain and another that combined CD28H, 2B4, and TCRζ cytoplasmic domains (**Figure 2A**). These five CD19 CARs were transfected into NKL.2DL1 cells, which have endogenous NKG2A and had been transduced to express KIR2DL1 (**Figure S1**). The three CD28H chimeras had a comparable cell surface expression, which was slightly lower than the two CD28 chimeras (**Figure 2B**). Killing assays with the HLA-I^-^ CD19^+^ 221 lymphoblast cell line as target cells showed that the CD19.NK-CAR carrying the three CD28H, 2B4, and TCRζ signaling domains induced the strongest NK-cell cytotoxicity toward 221 cells (**Figure 2C**). The third-generation T-cell CAR (denoted as T-CAR) and the CD28H-TCRζ CAR were intermediate, while the CD28-TCRζ and TCRζ alone did no better than untransfected NKL.2DL1 (**Figures 2C, D**). The resistance conferred by the CAR constructs to inhibition mediated by the NKG2A receptor for HLA-E and the KIR2DL1 receptor for HLA-C was tested with transfected 221 cells that express HLA-E or HLA-C. The CD19.NK-CAR was most efficient at overcoming inhibition by NKG2A or KIR2DL1, as NKL.2DL1 cells that expressed it lysed the HLA-I^+^ lymphoblast target cells (**Figure 2C**). The CD28H-TCRζ chimera induced partial protection from inhibition and the CD28-TCRζ and TCRζ alone provided no or very little enhancement of target cell lysis (**Figure 2C**). The T-CAR induced much less cytotoxicity than the CD19.NK-CAR under conditions of inhibition by NKG2A or KIR2DL1 (**Figure 2D**).

**Figure 3:**
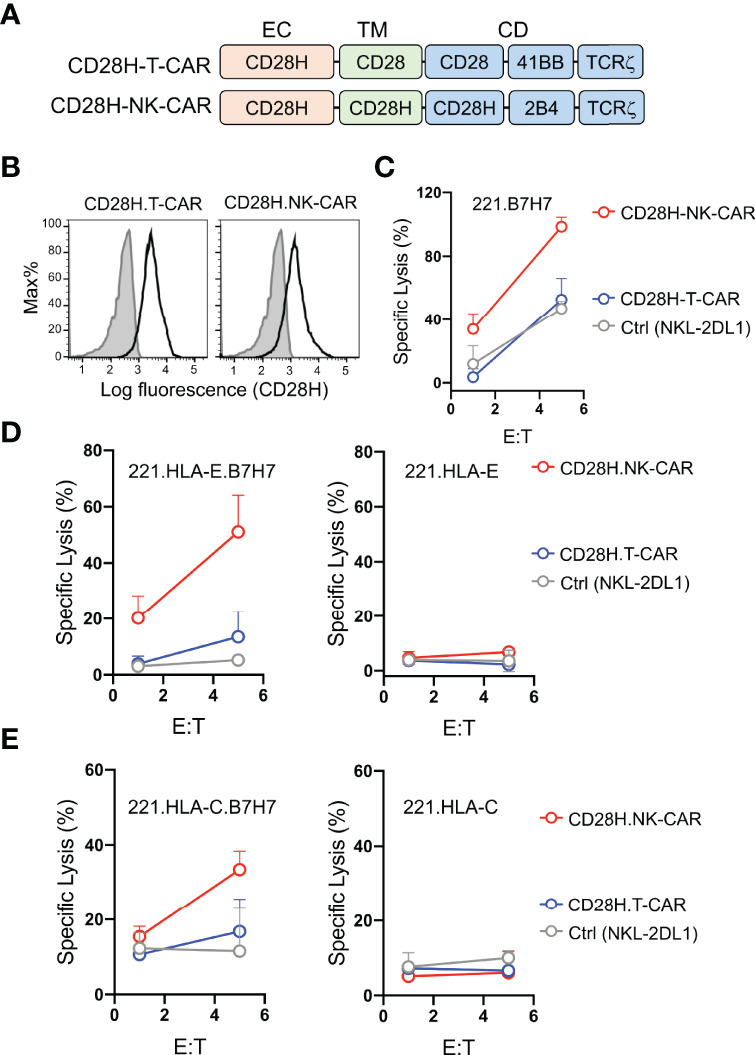
Lysis of B7H7^+^ HLA-I^+^ tumor cells by NK cells that express CD28H-CARs. **(A)** Schematic diagram of CAR constructs consisting of the extracellular domain of CD28H and the indicated combinations of signaling domains. **(B)** Flow staining to determine expression of CD28H.T-CAR, CD28H.NK-CAR and CD28H.CAR3 in NKL.2DL1 cells. **(C)** Lysis of 221.B7H7 cells by NKL.2DL1 expressing CD28H. CARs. **(D)** Killing assays using CAR-transduced NKL.2DL1 cells as effector and 221.HLA-E cells or B7H7-transfected 221.HLA-E cells as targets (n=2). **(E)** Lysis of 221.HLA-C (Cw15) cells or B7H7-transfected 221.HLA-C (Cw15) cells by NKL.2DL1 cells expressing CD28H.CARs (n=2).

B7H7 is highly expressed in certain tumors and can be potentially targeted by CARs (8). Therefore, we tested whether the CD28H receptor linked to chimeric signaling domains could be used to directly target B7H7^+^ tumor cells. We engineered a T-CAR and an NK-CAR by attaching the transmembrane (CD28) and cytoplasmic domains (CD28-41BB-TCRζ) of the CD19-T-CAR, and the transmembrane (CD28H) and cytoplasmic domains (CD28H-2B4-TCRζ) of the CD19-NK-CAR to the extracellular domain of CD28H (**Figure 3A**), and expressed them in NKL-2DL1 cells (**Figure 3B**). As noted with the CD19 CARs, the T-CAR with the CD28 transmembrane domain had a higher cell surface expression than its NK-CAR counterpart with the CD28H transmembrane domain. In the absence of inhibitory signaling, the lysis of 221-B7H7 cells by NKL-2DL1 cells was enhanced by the CD28H-NK-CAR but not by the CD28H-T-CAR (**Figure 3C**). Expression of HLA-E or HLA-C on 221 cells completely blocked lysis by NKL-2DL1 (**Figures 3D, E**). As expected, expression of the CD28H CARs, for which there was no ligand on 221 cells, on NKL-2DL1 did not induce a cytotoxic response (**Figures 3D, E**). However, coexpression of B7H7 with HLA-E or HLA-C on 221 cells rendered them sensitive to NKL-2DL1 cells that expressed the CD28H-NK-CAR (**Figures 3D, E** and **Figure S2**). By comparison, the CD28H-T-CAR induced a lower cytotoxic response toward 221-HLA-E-B7H7 and 221-HLA-C-B7H7 (**Figures 3D, E**). We conclude that a CD28H-based CAR expressed in NK cells is a viable option for targeting B7H7+ tumors.

**Figure 4:**
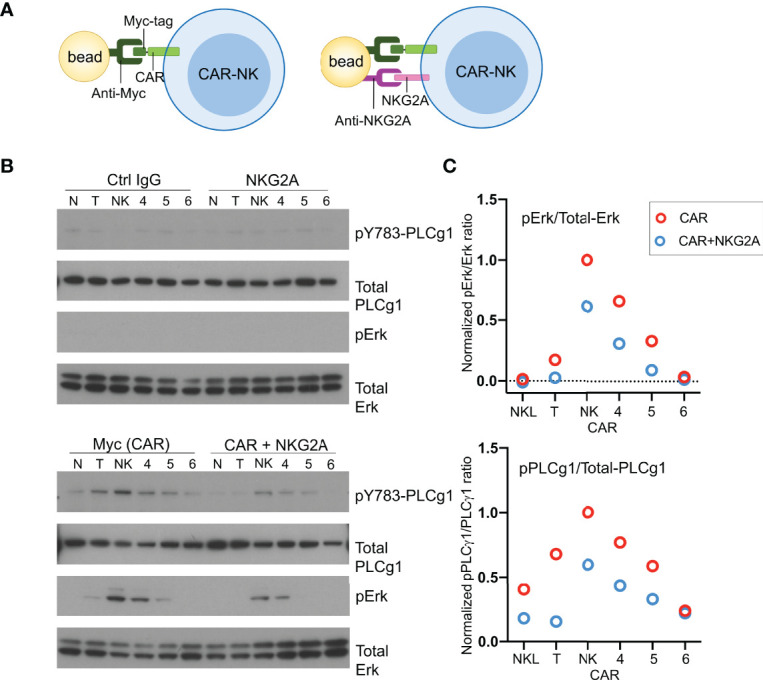
CD19.NK-CAR overcomes NKG2A-mediated inhibition. **(A)** Diagram of CAR stimulation by antibodies immobilized on beads. **(B)** Western blot detecting activation signals in lysates of CARs-expressing NKL cells after stimulation by beads. Untransfected NKL cells (N) were included as control. The indicated antibodies were pre-bound to beads, and then incubated with CAR-NK cells for 10 min. Cell lysates were probed for the indicated signaling molecules. **(C)** Western blot quantification of the pErk/total-Erk and pPLCγ1/total-PLCγ1 ratios (n=2).

To examine activation signals transduced through CARs, NKL cells expressing CD19-CARs were stimulated by myc-tag antibody coupled to beads (**Figure 4A**). Multivalent engagement of the N-terminal myc-tags induced phosphorylation of phospholipase C (PLC)-γ1 at Tyr783 and of the mitogen-associated protein kinases Erk1/Erk2 (*MAPK3*/*MAPK1*), which was not observed with control IgG coupled to beads (**Figure 4B**). After normalization for protein loading, the strongest phosphorylation of PLC-γ1 observed was that stimulated by the NK-CAR, followed by the T-CAR, CAR4 and CAR5 (**Figure 4C**). A different hierarchy was observed for Erk1/2 phosphorylation, which was strongest with NK-CAR, followed in decreasing order by CAR5, CAR5, and T-CAR (**Figure 4C** and **Figure S3**). The CAR6, which carries the cytoplasmic domain of TCRζ alone, did not induce phosphorylation of either PLC-γ1 or Erk1/2. Signaling by CARs in the presence of inhibitory signaling by NKG2A was tested by coupling antibodies to both CAR and NKG2A with the same beads. Beads carrying antibody to NKG2A alone did not induce phosphorylation of PLC-γ1 or Erk1/2, above that observed with control IgG (**Figure 4B**). Phosphorylation of PLC-γ1 by T-CAR was completely blocked by NKG2A co-engagement. In decreasing order, resistance to inhibition by NKG2A was observed with NK-CAR, CAR4, and CAR5 (**Figure 4C**). Similarly, phosphorylation of Erk1/2 was most resistant to inhibition after stimulation by NK-CAR, followed by CAR4 (**Figure 4C**). Overall, NK-CAR, and CAR4 to a lesser extent, induced activation signals that were resistant to inhibition by NKG2A.

## Discussion

Although downregulation of HLA-I expression occurs in some tumor cells, most tumor cells express sufficient HLA-I to inhibit NK cells. Chronic inflammation in tumors may also result in induction of HLA-I expression by IFN-γ and resistance to NK cells (14). In the context of bone marrow transplantation, a KIR–HLA mismatch may prove beneficial, as it avoids inhibition of NK cells and favors their graft-versus-leukemia function (15). In this scenario, T cells are depleted from the graft to reduce GVHD, and a graft from a family member who is identical for one HLA haplotype and mismatched for the other (haploidentical) is preferred. Grafted NK cell subsets for which HLA ligands for KIRs are missing in the host, bypass inhibition and exert graft-versus leukemia activity (15). However, allogeneic transplantation of hematopoietic stem cells is not a practical option to treat cancer. The high proportion of NK cells that express NKG2A, which increases further after transplantation or *ex vivo* expansion (16), and the upregulation of HLA-E in certain tumors (17–21), emphasize the need to develop strategies to overcome inhibition of NK cells for use in cancer immunotherapy.

The basis for tumor cell resistance to attack by NK cells varies. Independently of engagement of NK cell inhibitory receptors by HLA-I on tumor cells, tumor resistance could be due to weak NK–target cell adhesion or to the absence of tumor cell ligands for receptors that trigger NK cell cytotoxicity. Studies have reported that some combinations of signaling domains in CARs can overcome tumor resistance, although the reason for tumor cell resistance against NK cell attack is often not known (22–28). A CD19-CAR expressed in NK cells induced killing of several B-lineage acute lymphoblastic leukemia (ALL) cell lines (24). NK cells from peripheral blood had been co-cultured with irradiated, transfected K562 cells that co-expressed 4-1BB ligand and IL-15 at the plasma membrane. A CD19-CAR linked to the cytoplasmic domain of TCRζ alone, but not that of DAP10, could overcome tumor cell resistance. Addition of the 4-1BB cytoplasmic tail to TCRζ for costimulation further enhanced NK cell lysis of ALL cells (24). Several carcinoma cell lines, with various amounts of HLA-I at the cell surface, were tested in a study with CAR-NK cells. With a CAR consisting of a HER-2-targeting scFv and a chimeric CD28-TCRζ signaling domain, CAR-NK cell responses to tumor cells did not correlate with HLA-I expression (25). Another CD19-CAR expressed in NK cells, which had either the 2B4 or the TCRζ signaling domain, could overcome the resistance of a CD19^+^ leukemia cell line from NK cell attack (22). Combination of the two signals in a fused 2B4-TCRζ intracellular domain further augmented NK cell cytotoxicity (22). With autologous leukemia cells, degranulation was observed only with NK cells that expressed a CD19-CAR carrying both 2B4 and TCRζ signaling domains (22). Although these results suggest that CAR-NK cells could overcome inhibition by inhibitory receptors for HLA class, it is also possible that the CARs provided activation signals that were missing in NK–tumor cell interactions.

In the context of inhibition mediated by the HLA-E-specific inhibitory receptor NKG2A and the HLA-C-specific inhibitory KIRs, we and others have shown that incorporation of certain signaling domains in CARs may overcome inhibition (8, 27). Specifically, we proposed that a combination of signaling domains from NK activation receptors that synergize with each other in CARs tailored for NK cells could be a strategy to render NK cells more resistant to inhibition (29). Here we show that an NK-tailored CAR, which incorporates the signaling domains of CD28H, 2B4, and TCRζ outperformed a third-generation T-CAR. This NK-CAR induced stronger cytotoxic activity than the T-CAR and could trigger activation signals that resisted inhibition. These CAR-NK cells could overcome inhibition by receptors for HLA-I and kill HLA-I^+^ cancer cells.

B7H7 is also a ligand for the inhibitory KIR3DL3 receptor, which is expressed on some T cells (30–33). This explains the observed inhibition of T cells by B7H7 expression on other cells (32–35). Whether KIR3DL3, when expressed, inhibits NK cell cytotoxicity is unknown. As it carries only a single immunoreceptor tyrosine-based inhibition motif (ITIM), it may be weaker than the HLA-I specific inhibitory receptors studied here (NKG2A and KIR2DL1), which have two ITIMs in the cytoplasmic tail.

## Data Availability Statement

The raw data supporting the conclusions of this article will be made available by the authors, without undue reservation.

## Author Contributions

XZ conceived the study, carried out the experiments, analyzed the data, and wrote the paper. EOL conceived and supervised the study, and wrote the paper. All authors contributed to the article and approved the submitted version.

## Funding

This research was supported by the Division of Intramural Research, National Institute of Allergy and Infectious Diseases, NIH.

## Conflict of Interest

The authors are co-inventors on a patent application (PCT number PCT/US2020/024985) entitled "CD28H Domain-Containing Chimeric Antigen Receptors and Methods of Use''.

## Publisher’s Note

All claims expressed in this article are solely those of the authors and do not necessarily represent those of their affiliated organizations, or those of the publisher, the editors and the reviewers. Any product that may be evaluated in this article, or claim that may be made by its manufacturer, is not guaranteed or endorsed by the publisher.

## Supplementary Material

Supplementary Figure 1 Histograms showing expression of KIR2DL1 and NKG2A on NKL.2DL1 cells.

Supplementary Figure 2 Histograms showing expression of HLA-C, HLA-E, and B7H7 on 221 cells transfected with one or two these molecules.

Supplementary Figure 3 Bead stimulation of NKL cells expressing the indicated CARs. Untransfected NKL cells (N) were included as control. Control IgG and antibodies to Myc-tagged CAR and to NKG2A were bound to beads, and then incubated with CAR-NK cells for 10 min. Cell lysates were probed for total and phosphorylated versions of Erk and PLCg1. Results were used for the quantification shown in **Figure 4C**.
