# Supplementary material for: NK Cells Equipped With a Chimeric Antigen Receptor That Overcomes Inhibition by HLA Class I for Adoptive Transfer of CAR-NK Cells
Source: Front Immunol. 2022 May 2;13:840844. doi: 10.3389/fimmu.2022.840844 (PMC9108249; doi:10.3389/fimmu.2022.840844)
Supplement: Supplementary Figure 1 — Histograms showing expression of KIR2DL1 and NKG2A on NKL.2DL1 cells. [file DataSheet_1.pdf]

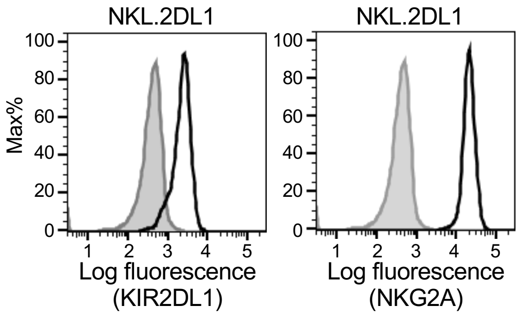

Supplementary Figure 1 Histograms showing expression of KIR2DL1 and NKG2A on NKL.2DL1 cells.

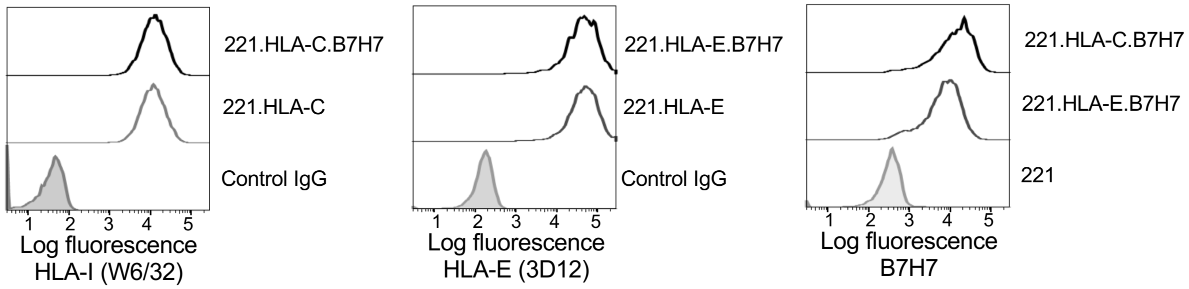

Supplementary Figure 2 Histograms showing expression of HLA-C, HLA-E, and B7H7 on 221 cells transfected with one or two of these molecules.

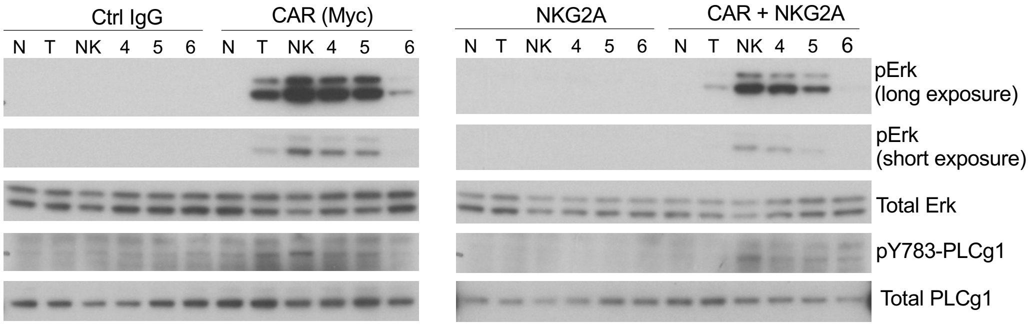

Supplementary Figure 3 Bead stimulation of NKL cells expressing the indicated CARs. Untransfected NKL cells (N) were included as control. Control IgG and antibodies to Myc-tagged CAR and to NKG2A were bound to beads, and then incubated with CAR-NK cells for 10 min. Cell lysates were probed for total and phosphorylated versions of Erk and PLCg1. Results were used for the quantification shown in Figure 4C.
